# A Single Bout of Moderate Aerobic Exercise Improves Motor Skill Acquisition

**DOI:** 10.1371/journal.pone.0141393

**Published:** 2015-10-27

**Authors:** Matthew A. Statton, Marysol Encarnacion, Pablo Celnik, Amy J. Bastian

**Affiliations:** 1 Motion Analysis Laboratory, Kennedy Krieger Institute, Baltimore, Maryland, 21205, United States of America; 2 Department of Physical Medicine and Rehabilitation, The Johns Hopkins School of Medicine, Baltimore, Maryland, 21205, United States of America; 3 Department of Neuroscience, The Johns Hopkins School of Medicine, Baltimore, Maryland, 21205, United States of America; The University of Western Ontario, CANADA

## Abstract

Long-term exercise is associated with improved performance on a variety of cognitive tasks including attention, executive function, and long-term memory. Remarkably, recent studies have shown that even a single bout of aerobic exercise can lead to immediate improvements in declarative learning and memory, but less is known about the effect of exercise on motor learning. Here we sought to determine the effect of a single bout of moderate intensity aerobic exercise on motor skill learning. In experiment 1, we investigated the effect of moderate aerobic exercise on motor acquisition. 24 young, healthy adults performed a motor learning task either immediately after 30 minutes of moderate intensity running, after running followed by a long rest period, or after slow walking. Motor skill was assessed via a speed-accuracy tradeoff function to determine how exercise might differentially affect two distinct components of motor learning performance: movement speed and accuracy. In experiment 2, we investigated both acquisition and retention of motor skill across multiple days of training. 20 additional participants performed either a bout of running or slow walking immediately before motor learning on three consecutive days, and only motor learning (no exercise) on a fourth day. We found that moderate intensity running led to an immediate improvement in motor acquisition for both a single session and on multiple sessions across subsequent days, but had no effect on between-day retention. This effect was driven by improved movement accuracy, as opposed to speed. However, the benefit of exercise was dependent upon motor learning occurring immediately after exercise–resting for a period of one hour after exercise diminished the effect. These results demonstrate that moderate intensity exercise can prime the nervous system for the acquisition of new motor skills, and suggest that similar exercise protocols may be effective in improving the outcomes of movement rehabilitation programs.

## Introduction

It is well known that regular physical activity has beneficial effects on cognitive function [1]. Specifically, long-term exercise training is associated with improvements in cognitive performance across domains including: attention and processing speed, long-term memory, and executive function [2]. Such changes in cognitive function may be explained by associations between cardiovascular fitness and increased cerebral blood flow [3], task-related brain activity [4], and brain volume [5].

Recently, it has also been shown that a single, acute bout of aerobic exercise is enough to lead to immediate improvements on cognitive learning and memory tests. For example, a single bout of aerobic exercise subsequently enhanced vocabulary learning and memory [6], attention and processing speed [7], and delayed free recall [8]. These “priming” effects, however, are likely due to different mechanisms than those underlying the effect of chronic physical activity on cognition, such as transient increases in neurochemical substances during a single exercise session [7, 6] which may, in turn, promote neuroplasticity [9, 10, 11].

While many have studied the effect of aerobic exercise on general cognitive function and declarative learning, less is known about the effect on motor learning. To date, only two studies have investigated the effect of acute aerobic exercise on motor learning tasks [12, 13]. Specifically, Roig et al. [12] showed that a short, high intensity bout of aerobic exercise performed either before or after learning improved retention, but not acquisition, of a visuomotor continuous cursor-tracking task. On the other hand, Mang et al. [13] additionally found that a similar high intensity exercise protocol could, in fact, improve one specific component of performance during acquisition (temporal precision), but had no effect on spatial accuracy during training.

These studies demonstrate a benefit of aerobic exercise on motor learning, yet there are many aspects of the exercise/motor learning relationship that still need to be explored. For example, varying intensities or durations of exercise may lead to different effects on motor learning tasks. While some studies have shown beneficial effects on cognitive function after short, high intensity bouts of exercise [6, 12, 13], evidence suggests that performing exercise at submaximal intensities for longer durations (i.e. 20–60 minutes) more consistently benefits cognitive performance [14, 8]. With this in mind, it is important to consider the influence that moderate intensity, longer duration exercise might have on motor learning performance compared with the short, high intensity exercise of the aforementioned studies. This is particularly important when thinking ahead for application to patient groups who may not be able to participate in high intensity activities.

Furthermore, the timing of motor learning in relation to exercise should be explored. The greatest benefit in cognitive function has generally been observed when exercise is performed immediately before, as opposed to during or after, learning [8]. That being said, it is not fully understood how long cognitive improvements may remain after exercise cessation. Kashihara and Nakahara [15] demonstrated improved reaction time during only the first 8 minutes after exercise, with subsequent decay back to baseline performance. On the other hand, Heckler and Croce [16] showed that addition and subtraction performance remained elevated for 15 minutes, and Joyce and colleagues [17] found persistent improvements in executive function up to 52 minutes after exercise was completed. Thus, introducing a period of rest in between exercise and motor learning may help distinguish whether acute exercise provides either an immediate or sustained change in performance.

Finally, subjects in the aforementioned exercise/motor learning studies [12, 13] were only judged on the accuracy of their cursor movements, while cursor speed was fixed. When learning a real-world motor skill, however, practice often involves continuously adjusting both movement speed and accuracy to master the task. Recent studies of motor skill learning have quantified changes in this speed-accuracy tradeoff function “to avoid falsely identifying improvements in speed while accuracy worsens (or vice versa) as learning” [18]. Fully understanding the effect of exercise on motor learning therefore requires consideration of this tradeoff between movement speed and accuracy during training.

Here we investigated how an acute, moderately intense bout of aerobic exercise affects motor skill learning. We asked how exercise might differentially affect two distinct components of motor learning performance: movement speed and accuracy. In experiment 1, we asked whether exercise would lead to immediate changes in motor skill acquisition, and whether any effect would decay if a long rest period was introduced between exercise and the motor learning session. In experiment 2, we investigated if effects persisted across training sessions on consecutive days, as well as how exercise affects both acquisition and retention of motor skill.

We hypothesized that performing a bout of moderate intensity exercise before motor learning would improve both the acquisition and retention of motor skill, while resting in-between exercise and learning would diminish this effect. Our results showed that participants engaging in aerobic exercise exhibited significantly greater motor acquisition, but no change in retention. This effect was driven by improved movement accuracy during training, whereas movement speed was unaffected. As we hypothesized, the beneficial effect of exercise was only apparent when motor learning occurred immediately after exercise.

## Materials and Methods

### Participants

We studied 46 healthy adults (16 M, 23 ± 3 years old). The experimental protocol was approved by the Johns Hopkins Medicine Institutional Review Board and conformed to the standards set by the *Declaration of Helsinki*. All participants provided written informed consent before testing, and were screened for cardiovascular risk factors in accordance with the American Heart Association and American College of Sports Medicine Health/Fitness Facility Pre-participation Screening Questionnaire [19]. Only subjects with fewer than two risk factors were considered eligible to participate in the aerobic exercise sessions. All subjects completed an additional questionnaire to report demographic data including age, height, weight, and general physical activity level. From this data, body-mass index (BMI) and average weekly metabolic equivalent (MET) expenditure values were calculated based on the Compendium of Physical Activities Tracking Guide [20].

### Motor learning task

**Fig 1:**
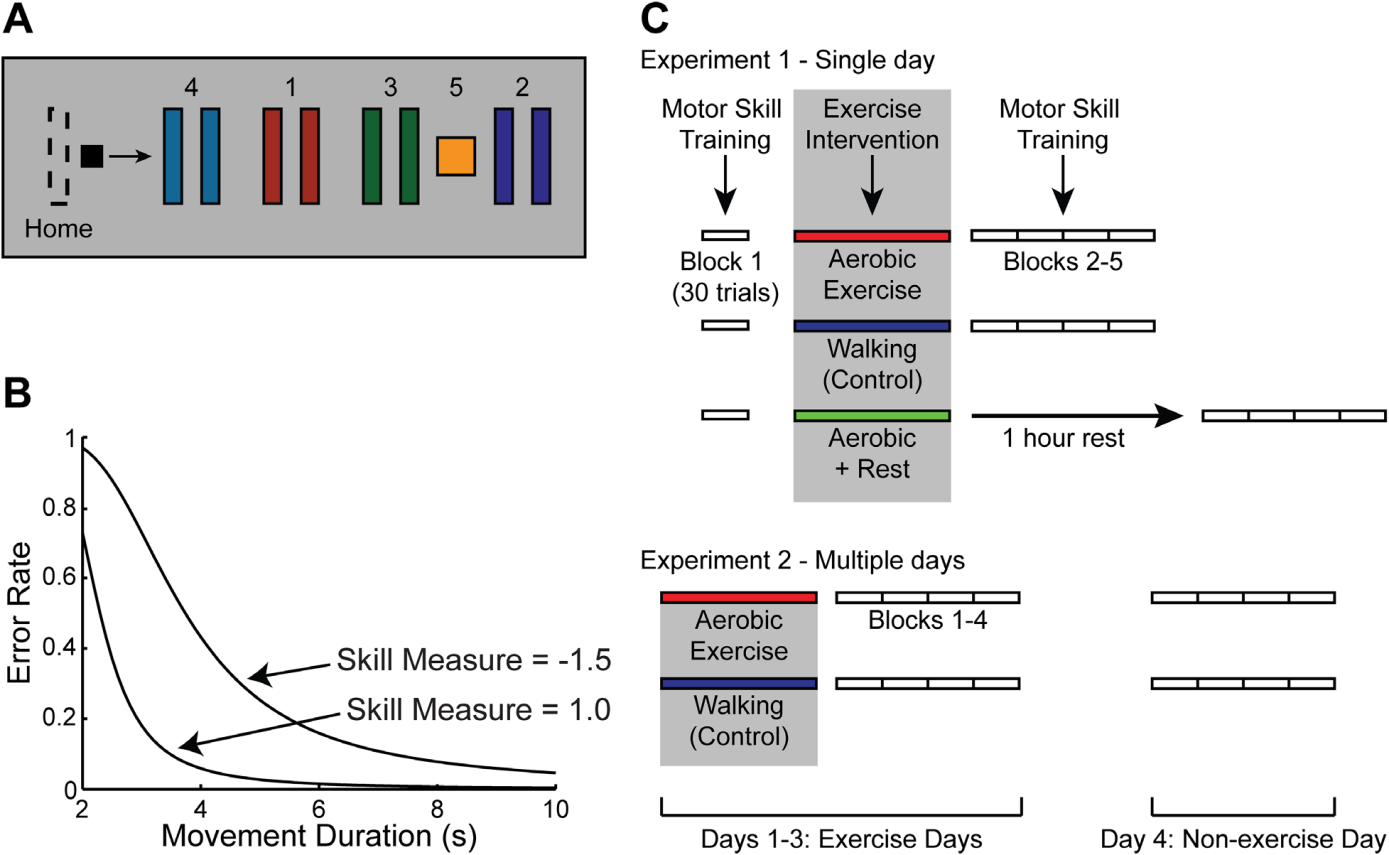
Motor learning task and experimental paradigm. (A) Sequential Visual Isometric Pinch Task (SVIPT). Participants were instructed to move the cursor (black square) as quickly and accurately as possible between the “Home” position and each of the five gates by varying the amount of force applied to a force transducer. (B) Sample skill measure change during SVIPT training, shown as a shift in speed-accuracy tradeoff function (SAF). Note that as movement duration and/or error rate improve, SAF exhibits a downward shift resulting in increased skill measure. (C) Overview of study design. Participants in Aerobic and Aerobic + Rest groups ran at 65–85% of their age-predicated maximum heart rate for 30 minutes either immediately (Aerobic) or one hour (Aerobic + Rest) prior to motor skill training. Participants in the Control groups walked at a slow pace (1.0 m/s) for 35 minutes immediately prior to motor skill training.

We used the Sequential Visual Isometric Pinch Task (SVIPT) to assess motor skill learning [21, 22, 23, 24, 18]. Subjects were seated in front of a computer monitor and held a force transducer between the thumb and index finger of the dominant hand. Isometrically squeezing the force transducer controlled the movement of an on-screen cursor. Participants were instructed to move the cursor as quickly and accurately as possible between a Home position and five gates by varying the force applied to the transducer (Fig 1A). Transduction of pinch force into cursor movement was logarithmic in order to increase the difficulty of the task. We defined movement duration per trial as the time from movement onset to reaching gate 5. Error rate was calculated as the proportion of trials with at least one overshooting or undershooting movement per block (block is the average across 30 consecutive SVIPT trials).

To quantify motor learning, we determined changes in the speed-accuracy tradeoff function (SAF) (Fig 1B). The proposed estimate of changes in the SAF, previously derived by Reis et al. [21], is the skill parameter, as follows:

$$\text{skill parameter = }\frac{\text{1-error rate}}{\text{error rate (ln}{\left(\text{movement duration}\right)}^{\text{b}}\text{)}}\text{,}$$

where error rate and movement duration are averages over 30 trials, and the value of b is 5.424. Improvements in movement duration and/or error rate can thus result in a shift of the overall speed-accuracy tradeoff function, reflected by an increase in the skill parameter, but improved movement duration alongside worsened error rate, or vice versa, would not result in increased skill. As described in previous studies, the noise in this skill parameter estimate is multiplicative, so we logarithmically transformed it to validly perform parametric statistical analyses. The natural logarithm of the skill parameter is referred to as the "skill measure" [21, 22].

To quantify between-day motor skill retention, we defined offline changes in skill measure as:

$$\text{Offline changes = }\overset{\text{\# days-1}}{\underset{\text{i = 1}}{\sum }}\left({\text{skill measure}}_{\text{i+1,block 1}}{\text{-skill measure}}_{\text{i, last block}}\right)$$

### Exercise intervention

Our exercise intervention involved subjects performing either an acute bout of moderate intensity aerobic exercise (running) or low intensity exercise (walking) on a treadmill (Woodway, Waukesha, WI). The running protocol began with a five minute warm-up period consisting of two minutes of walking at 1.0 m/s followed by three minutes of running. During the running warm-up, treadmill speed was adjusted until the subject's heart rate reached 65–85% of their age-predicted maximum heart rate. We designed the exercise protocol with this heart rate range to be consistent with previous studies demonstrating a cognitive benefit from this exercise intensity [8]. Following the five minute warm-up period, subjects ran for 30 minutes. Treadmill speed was adjusted as needed during running to ensure heart rate stayed within the desired range. The walking protocol consisted of 35 minutes of walking at 1.0 m/s. Heart rate was monitored continuously during exercise via telemetry (WearLink 31 transmitter, Polar Electro, Kempele, Finland). Heart rate, treadmill speed, and perceived exertion (based on the Borg Rating of Perceived Exertion scale [25]) were recorded every five minutes.

### Experimental design

#### Experiment 1 –single day

Fig 1C shows a schematic describing Experiments 1 and 2. Participants in the single-day experiment performed one baseline block of 30 trials of SVIPT training then were assigned to one of three groups that performed different exercise interventions. The Aerobic and Aerobic + Rest groups performed the aforementioned running protocol either immediately (Aerobic) or one hour (Aerobic + Rest) before four additional blocks of motor training. Subjects in the Aerobic + Rest group sat quietly during the one hour rest period. The Control group performed the walking protocol immediately before the four blocks of motor training.

#### Experiment 2 –multiple days

Participants in the multiple-day experiment were divided into two groups: Aerobic or Control. All subjects performed four blocks of SVIPT training on each of four consecutive days. On days 1–3 (exercise days), the Aerobic group performed the running intervention immediately before four blocks of motor training, while the Control group performed the walking intervention. On day 4 (non-exercise day), participants in both groups performed four blocks of motor training without any exercise intervention.

### Data analysis

Analysis of movement durations and errors per trial was semi-automated using a custom Matlab script (ForceDataViewer, see Reis et al. [21]). In experiment 1, demographic data were compared using a 1-way analysis of variance (ANOVA) to assess any differences between groups in age, gender, or physical fitness/activity levels (BMI, MET-hrs/week) which may have influenced performance on the motor learning task. Skill measure, error rate, and movement duration were each analyzed with a repeated-measures ANOVA to assess group differences across blocks (5 blocks). Post-hoc analysis was performed using Tukey’s HSD test when applicable to examine differences between groups in overall performance and within each block. In experiment 2, demographic data were compared using independent samples t-tests. Statistical analysis of motor learning was performed separately for exercise days (days 1–3) and the non-exercise day (day 4) to differentiate changes in motor learning and retention immediately after exercise versus residual changes when exercise was no longer performed. For exercise days, separate repeated-measures ANOVAs were performed for skill measure, error rate, and movement duration to compare group differences across days (3 days) and blocks (4 blocks per day). For the non-exercise day, repeated-measures ANOVAs were performed for the same parameters to compare group differences across blocks (4 blocks). Lastly, independent samples t-tests were used to analyze offline changes in skill measure, error rate, and movement duration within exercise days and between exercise and non-exercise days. For all analyses, we used the Greenhouse-Geisser correction when sphericity was violated and report the uncorrected degrees of freedom together with the Greenhouse-Geisser epsilon.

## Results

### Participant demographics

A total of 46 subjects completed this study (Experiment 1: n = 24, Experiment 2: n = 22). Two participants were excluded from Experiment 2 because they reported no baseline physical activity level. 44 subjects were thus included in the final analysis. No significant differences between groups were found in demographic data in either experiment (Table 1).

**Table 1:** 

Demographics							
	Experiment 1—Single Day				Experiment 2—Multiple Days		
	Aerobic	Control	Aerobic + Rest	p-value	Aerobic	Control	p-value
No. of subjects	8	8	8		10	10	
Age (years)	22.4 ± 1.1	22.0 ± 1.0	21.1 ± 1.1	.690	24.9 ± 1.1	22.4 ± 0.9	.093
Gender (M:F)	3:5	3:5	2:6	.848	4:6	4:6	1.00
Body-mass index	21.4 ± 0.7	23.0 ± 1.2	20.1 ± 0.6	.095	23.0 ± 0.8	22.9 ± 0.8	.930
MET-hrs/week	23.5 ± 5.3	32.9 ± 6.1	35.1 ± 9.5	.492	25.8 ± 4.6	22.2 ± 5.2	.618

Note: Data shown as group mean ± SEM.

### Exercise intervention

The aerobic exercise protocol resulted in an average heart rate of 165 (± 10) beats/min during exercise, which corresponds to 84.0% (± 3.9%) of each subject’s age-predicted maximum heart rate. Individuals subjectively reported this exercise intensity as 13 (± 2) on the Borg Rating of Perceived Exertion scale [25], which indicates a “somewhat hard” level of exertion. On the other hand, subjects performing the walking protocol had an average heart rate of 92 (± 12) beats/min and reported perceived exertion of 7 (± 1) on the Borg scale—indicating “extremely light” exertion.

### Motor learning results

#### Single day motor acquisition

**Fig 2:**
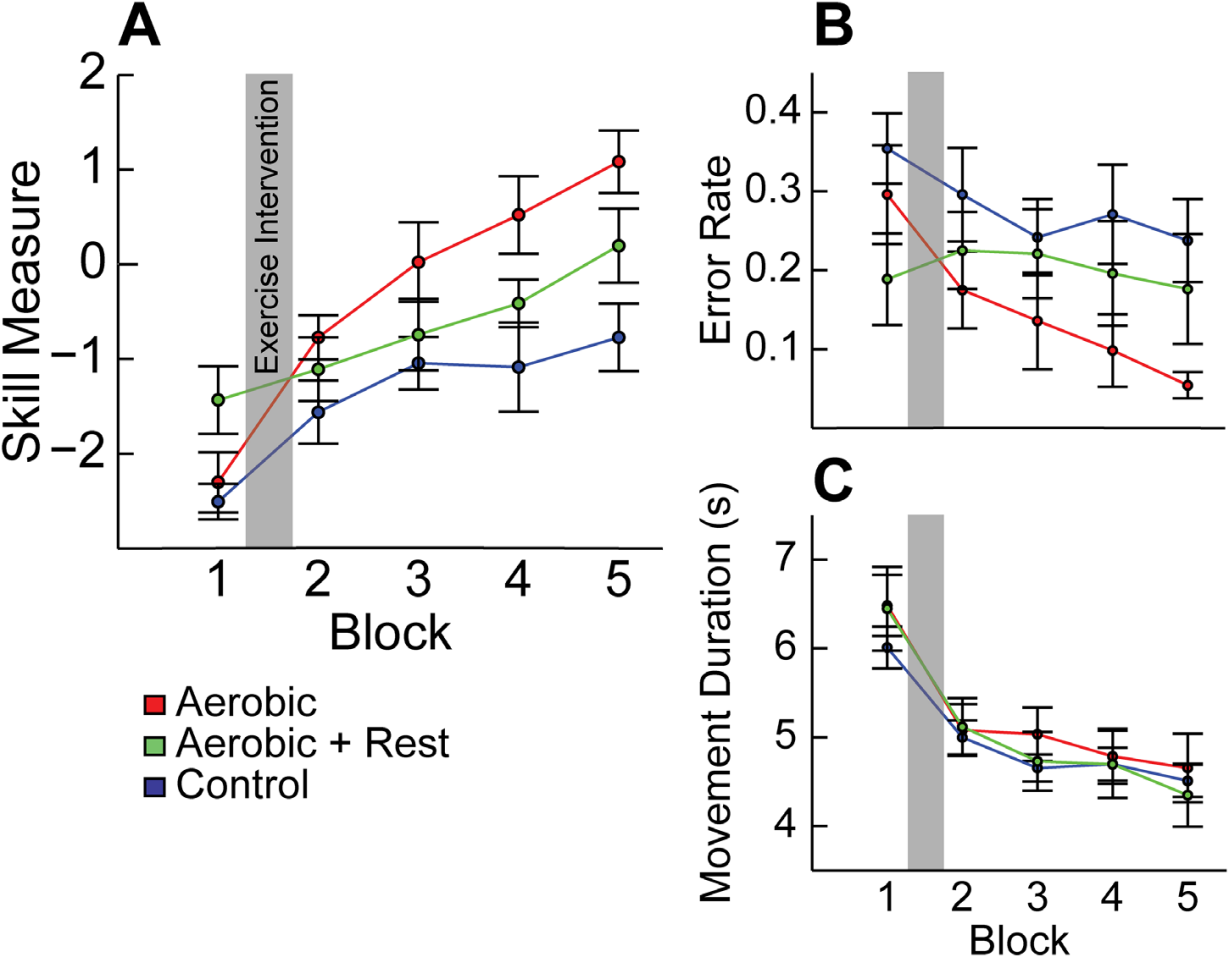
SVIPT performance for single day experiment. Exercise intervention, shown as a grey bar, occurs between blocks 1 and 2 of SVIPT training. (A) Learning curve during motor training. Repeated-measures ANOVA revealed a significant main effect of Group (p = .021) and a Block x Group interaction (p = .007) for skill measure, indicating improved motor acquisition in the Aerobic group when exercise was performed immediately before motor skill training. (B) Error rate and (C) movement duration. Results indicate that the effect of exercise on skill measure may have been driven by a non-significant improvement in error rate (p = .127), as opposed to movement duration (p = .828). All data shown as mean ± SEM.

Learning curves for Experiment 1 are shown in Fig 2A. Over the course of motor skill training on a single session, all groups showed significant improvement in skill measure (Block: F [4, 84] = 34.47, p < .001). Our ANOVA also revealed a significant main effect of Group (F [2, 21] = 4.656, p = .021) and a Block x Group interaction on skill measure (F [8, 84] = 2.850, p = .007). Consistent with our hypothesis, post-hoc analysis showed that the Aerobic group performed better than Control subjects (p = .017), but the Aerobic + Rest group did not (p = .165). These results indicate that aerobic exercise performed immediately before SVIPT training increased motor acquisition, but resting for one hour after completing exercise diminished this effect. We should note that post-hoc analysis also revealed that the Aerobic + Rest group demonstrated a difference in baseline (block 1) performance compared with the Control group (p = .046). Despite this initial offset, only the Aerobic group was able to reach a significantly higher skill level than the Control group by the end of the training session (block 5: p = .004).

Differences in skill measure could be driven by changes in error rate (Fig 2B), movement duration (Fig 2C), or a combination of both. Our results indicate that the improvement in skill measure may have been driven primarily by a non-significant improvement in error rate (p = .127), rather than movement duration (p = .828).

#### Multiple day motor acquisition

**Fig 3:**
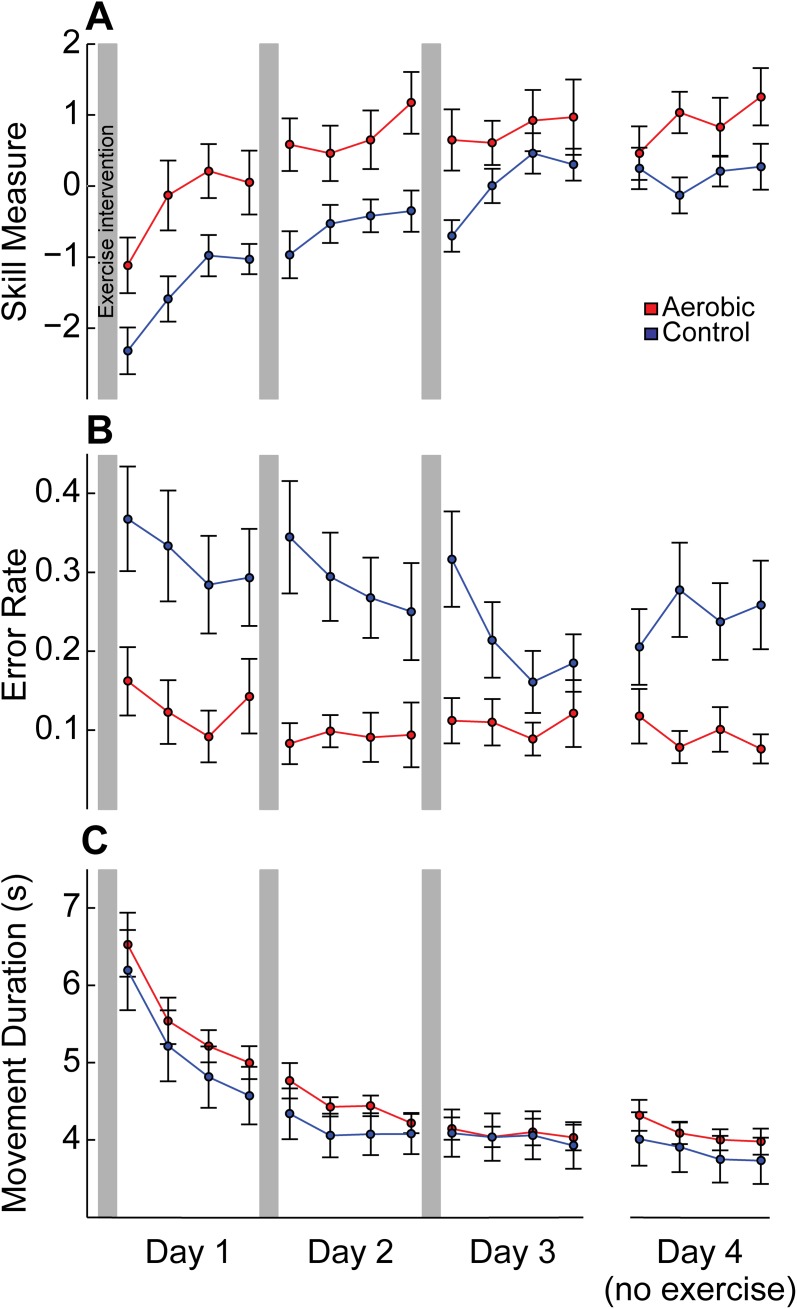
SVIPT performance for multiple day experiment. Experimental sessions took place on four subsequent days. Exercise intervention was performed immediately prior to SVIPT training on days 1–3, and only SVIPT training was performed on day 4. (A) Learning curve across all four days. Aerobic exercise prior to motor training led to an immediate and persistent improvement in skill measure compared with Controls (Group: p = .011) throughout exercise days. On the non-exercise day, Aerobic subjects tended to continue to exceed Controls in both overall performance (Group: p = .081) and learning rate (Block x Group: p = .065). No significant offline changes (i.e. retention) of skill measure were found for either exercise or non-exercise days. (B) Error rate and (C) movement duration. On exercise days, results indicate that the exercise intervention led to a significantly lower error rate in the Aerobic group (p = .006), but had no effect on movement duration (p = .479). A Group effect (p = .013) and Block x Group interaction (p = .022) on error rate were found on the non-exercise day as well. No significant offline changes in error rate or movement duration were found for either exercise or non-exercise days. All data shown as mean ± SEM.

Subjects in Experiment 2 exercised prior to performing the motor skill task on the first three days and performed only the motor skill task on the fourth day. Learning curves are shown in Fig 3A. ANOVA for skill measure on exercise days (days 1–3) showed significant effects of Block (F [3, 54] = 13.462, p < .001, ε = .646), Day (F [2, 36] = 53.308, p < .001, ε = .676), and most importantly, Group (F [1, 18] = 8.128, p = .011), indicating that both groups experienced significant skill learning throughout training, but the Aerobic group performed significantly better than the Control group overall. Unlike the single-day experiment, however, there were no significant interactions between group and time (Block x Group: p = .423; Day x Group: p = .121). Instead, aerobic exercise led to an overall improvement in performance throughout each training session.

We found that the overall increase in skill measure in the Aerobic group was specifically driven by improved error rate (Group: F [1, 18] = 9.935, p = .006; Fig 3B) throughout training, as opposed to movement duration (Group: F [1, 18] = .524, p = .479; Fig 3C). Thus, while greater skill could be achieved by improving speed and/or accuracy, subjects performing aerobic exercise chose the same movement speed as their walking counterparts but were able to perform the task with significantly greater accuracy.

Additionally, our ANOVA revealed that the Control group decreased their errors faster than the Aerobic group within each training session on exercise days, as demonstrated by a Block x Group interaction on error rate (F [3, 54] = 3.644, p = .031, ε = .735). In other words, although subjects in the Aerobic group made fewer overall errors during motor training, the Control group demonstrated a faster *rate* of error reduction within training sessions.

All subjects returned for an additional non-exercise day to determine whether exercise-induced effects on motor learning were state-dependent (i.e. requiring a preceding exercise session) or could be expressed when exercise was no longer performed beforehand. For the non-exercise day, ANOVAs revealed a significant main effect of Group (F [1, 18] = 7.669, p = .013) and a Block x Group interaction on error rate (F [3, 54] = 3.490, p = .022), as well as trends towards both a main effect of Group (F [1, 18] = 3.422, p = .081) and a Block x Group interaction on skill measure (F [3, 54] = 2.557, p = .065). In other words, subjects who had previously trained following aerobic exercise continued to perform better than Control subjects without the need for continued exercise sessions.

#### Motor skill retention

Skill retention was evaluated in terms of offline changes in skill measure, error rate, and movement duration. The two groups did not exhibit significant differences in offline changes for any parameter either on exercise days (skill measure: p = .699, error rate: p = .091, movement duration: p = .823) or between the exercise and non-exercise days (skill measure: p = .270, error rate: p = .525, movement duration: p = .262). We conclude that aerobic exercise improved motor learning during training sessions, but did not change retention in between days.

## Discussion

The goal of this study was to determine the effect of acute bouts of moderate intensity aerobic exercise on the acquisition and retention of a new motor skill. We found that aerobic exercise led to significantly greater performance of the motor learning task during training sessions, but had no effect on between-day retention. This beneficial effect, however, was dependent on motor training occurring immediately after exercise—resting for a period of one hour diminished the effect. Importantly, although aerobic exercise did not affect the amount of retention, it led to continued improvement of online motor skill performance on subsequent days when exercise was no longer performed beforehand.

The SVIPT task and skill measure allowed us to assess motor skill learning while accounting for changes in both speed (i.e. movement duration) and accuracy (i.e. error rate). By enabling subjects to freely adjust both components during training, we could determine which component, if any, was more affected by exercise. We found that increased motor skill in the Aerobic groups was primarily driven by improved movement accuracy, rather than speed. Whether this increase in performance represents a benefit of exercise on learning, however, is somewhat ambiguous. This distinction between performance and learning is important, as changes in short-term performance during training do not necessarily indicate long-term learning [26]. It is possible that exercise provided an initial boost in performance that merely persisted during the remainder of each training session. However, if the exercise intervention simply improved motor performance without producing a lasting change in skill (i.e. increased performance without learning), we would expect subjects in both groups to perform similarly on the non-exercise day of Experiment 2. In contrast, subjects who had previously trained after aerobic exercise continued to perform better than their walking counterparts, indicating that greater motor skill achieved after exercise was encoded and stored. This suggests that moderate aerobic exercise can promote the acquisition of a new motor skill. However, future studies are necessary to help distinguish whether exercise facilitates learning directly (by specifically affecting motor learning processes) or indirectly (by increasing performance during training).

There are several possible explanations for the benefit of acute exercise on learning and motor performance. For instance, psychological models contend that exercise leads to increased arousal and cognitive resource allocation, resulting in improved performance on cognitive tasks [27, 28, 29]. Indeed, moderate autonomic arousal, even in the absence of exercise, has been shown to improve motor performance [30] which could in part explain the results of the present study. In contrast, neuroendocrinological theories attribute learning improvements to exercise-induced release of neurotransmitters, hormones, and other neuromodulatory substances that support cognition [31, 32]. Exercise is known to result in changes in circulating levels of numerous neurochemical substances including epinephrine, dopamine, serotonin, brain-derived neurotrophic factor (BDNF), and cortisol [31, 32, 33]. It was recently shown that BDNF, norepinephrine, and lactate may be important mediators of the effect of *high* intensity exercise on motor learning [34], however it has yet to be determined which biomarkers may underlie the effect of moderate intensity exercise on motor acquisition. Though psychological and physiological effects of exercise on cognition are likely interrelated, our observation that resting for one hour after exercise negated the effects supports the notion that the exercise/learning relationship is mediated by neuroendocrine factors. Studies of the time-course of catecholamine and BDNF changes after exercise have shown elevations in circulating levels for up to 60 minutes after exercise is completed [33, 6, 35]. Our finding, then, may demonstrate the transient consequences of the short-lasting elevations of these substances. While motor acquisition was enhanced immediately after exercise, it is plausible that resting for 60 minutes allowed substances such as norepinephrine and BDNF to return to baseline levels, thus diminishing the beneficial effect of the exercise session.

The effects of aerobic exercise on motor learning are likely dependent upon both the intensity and timing of exercise in relation to learning. It has previously been suggested that exercise affects the molecular mechanisms responsible for memory encoding (i.e. the acquisition of information during exposure) and consolidation (i.e. the transfer of previously encoded information to long-term memory, reflected in retention tests) in a time-dependent manner [36, 37]. In theory, a bout of exercise performed before learning would mainly influence memory encoding [36, 8, 6], as seen in our results, whereas exercise performed after exposure would prime consolidation and retention mechanisms [36, 38]. However, recent findings by Roig et al. [12] contradict these ideas as high intensity exercise improved motor skill retention, but not acquisition, whether exercise was performed before *or* after learning. It may be surprising that exercise before learning had the same effect on retention as exercise after learning, yet exercise-induced release of neurochemicals is largely intensity-dependent [6,7]. Therefore, the findings of Roig et al. [12] may indicate the persistence of neurophysiological changes after high intensity exercise that last long enough to influence the initial stages of memory consolidation [36]. However, the moderate intensity exercise used in the present study, although beneficial to memory encoding during motor acquisition, may not have been enough to elicit the lasting change necessary to influence memory consolidation processes.

Though retention was not specifically improved by our exercise intervention, the fact that increased motor skill achieved after exercise was retained between days is important in itself. Previous studies have shown that between-day motor skill retention is not a guarantee, as different interventions can either disrupt [23, 24] or improve [21] these retention mechanisms. The effects of our exercise protocol were restricted to online performance during training sessions, yet the ability of Aerobic subjects to retain this increased performance level on subsequent days, whether or not exercise was performed beforehand, supports the notion that moderate aerobic exercise can be an effective means to facilitate lasting improvements in motor skill.

Previous work on exercise and motor learning assessed performance solely based on movement accuracy. This work showed that high intensity exercise primarily improved motor skill retention [12, 13], with one study additionally demonstrating a benefit in temporal precision during acquisition but no effect on spatial accuracy [13]. If we compare this to our findings where speed and accuracy were both considered, yet exercise significantly increased accuracy, we might have expected an even greater benefit in acquisition in the simpler, “accuracy-only” tasks of the previous studies. This discrepancy in the results may be attributed to differences in exercise intensity. Long-duration exercise leading to dehydration [39] and intense exercise leading to fatigue [40] can have either deleterious or null effects on cognitive function. Furthermore, while increased neurochemical substances due to exercise may be beneficial to memory formation, an excess of certain substances may actually impair motor performance. Highly elevated levels of epinephrine, for instance, are associated with decreased fine motor control and increased physiological tremor [41]. For this reason, β-adrenergic receptor blockers are often used to improve performance in sports requiring movement precision [42]. Our results may suggest that exercise intensity should be tempered to optimize the benefit during motor acquisition. While a single bout of aerobic exercise can facilitate motor acquisition through improved movement accuracy, we speculate that fatigue, dehydration, or an overabundance of certain neurochemical substances following high intensity exercise may mask this effect.

One unexpected finding of the current study was the difference in within-day error rate reduction between groups in Experiment 2: while the Aerobic group had lower overall error rate during motor training, the Control group reduced their error rate more quickly within training sessions on exercise days. One possible explanation for this result is that performance of the aerobic exercise protocol had already improved overall error rate to near-asymptotic levels in the Aerobic group. In other words, the Control group simply had more room for improvement. In future experiments, increasing the difficulty of the task (e.g. by applying a more complex visuomotor transformation between applied pinch-force and cursor movement) may help elucidate the effect of aerobic exercise on within-day error rate reduction by allowing more room for continued improvement. In the present study, however, we can conclude that aerobic exercise led to improved error rate throughout all motor training sessions.

## Conclusions

We have shown that a single bout of moderate intensity aerobic exercise can facilitate motor skill acquisition. This result could serve important purposes in the context of rehabilitation. For example, functional recovery from hemiparesis after stroke is dependent in part on motor learning processes, for instance to learn compensatory movements to accomplish task goals [43]. One recent study demonstrated that long-term aerobic exercise training in chronic stroke patients (3 sessions per week for 8 weeks) led to improved performance on two different motor learning tasks—a serial reaction time task and a precision grip task [44]. Our results suggest that a single bout of moderate intensity aerobic exercise, performed immediately prior to rehabilitation sessions, may be enough to prime patients for improved motor acquisition as well, thus facilitating success in movement rehabilitation programs without the confounding neural noise or excessive arousal which may be introduced by high intensity exercise. Further, it may be more feasible to implement a moderate intensity program in many patient groups for which high intensity training is challenging. Importantly, it remains to be determined which neurochemical substances or neurophysiological processes underlie the effect of moderate aerobic exercise on motor learning. Understanding these basic mechanisms will be essential to prescribing the most effective exercise regimen for the acquisition and retention of new motor skills.
